# Big (pig) data and the internet of the swine things: a new paradigm in the industry

**DOI:** 10.1093/af/vfz002

**Published:** 2019-04-11

**Authors:** Carlos Piñeiro, Joaquín Morales, María Rodríguez, María Aparicio, Edgar García Manzanilla, Yuzo Koketsu

**Affiliations:** 1 PigCHAMP Pro Europa SL, C/ Dámaso Alonso, Segovia, Spain; 2 Teagasc, Pig Development Department Moorepark, Fermoy, Ireland; 3 School of Agriculture, Meiji University, Kawasaki, Japan

**Keywords:** decision making, information systems, pig data, predictive analytics, swine health and production

**Implications**

- Big data collected on farms can be transformed into useful information to improve decision making and maximize productivity. A swine management system consisting of tools (software and devices), with a protocol and standard operative procedures, can generate the necessary information for the decision-making process.
- New technologies such as electronic feeders and artificial intelligence systems capturing big data will provide a better understanding of animal requirements and behavior, increasing efficiency and sustainability.
- Biosecurity can be improved using tracking devices for farm staff, recording movements real-time to decrease disease risks and consequently, improve health and productive performance.

## Introduction

The present work addresses the use of data in improving decision making and farm productivity, one of the aspects that has generated more interest in swine production in recent years. In the current review, the limitations of data management and recently developed strategies in this sector have been revised, together with the need for new technologies and their use in the evolution of the precision livestock farming concept. The importance of traditional animal-oriented data together with environment-oriented data are stressed.

## Limitations of Current Data Management in Swine Production

In the last three decades, the data used by farmers has been limited. Most of the activities were basic and mainly focused on the management of farm tasks, with limited capacity for analysis. They were focused on sow reproductive data, which consisted of collecting data on mating, farrowing, and weaning to generate working lists (sows to wean, farrow, or mate; pregnancy checks; and vaccinations) or basic production summaries. These, in a best-case scenario, included the impact of certain explanatory variables such as parity, weaning to estrus interval or repeat percentages among others. In post-weaning (nursery-grow-finishing), the most common reports used were body weight, feed intake, feed efficiency, and mortality by batch. Integration of data from different sources (abattoir, laboratory, reproduction, health, or medicine use) was difficult and rare (MAPAMA, 2019) and therefore of little value to generate knowledge for strategic decision making. Another improvable aspect is the limited amount of or lack of data support services that generates value and promotes digital transformation in the swine sector.

The use of data in agricultural crops has increased exponentially in recent years. However, its use in livestock is still limited. In pigs, data collection has not changed for many years and analysis is still focused on the main reproductive key performance indicators such as farrowing rate, the number of repeat services, total born piglets, born alive, stillborn, mummifies, weaning to first service interval, and pre-weaning mortality. Other types of data, such as environmental or slaughterhouse, or data from feeding stations have not been used in practice except to create simple alerts, such as detection of temperatures out of range or sows that have not eaten. Among the reasons for this slow progress are the low added value perceived by producers, the good margins that for years prevented the need for improvement based on data analysis, the scarcity of professionals with a solid comprehensive education of farm data management or the lack of tools adapted to the sector to facilitate the process of extracting value, benchmarking, and monitoring. In addition to these issues, companies manufacturing farm equipment and software that generate data did not facilitate its extraction and use.

Most producers use some sort of management software for basic management tasks but do not use data to its full potential. This software should be only one part of an integrated information system. Most software programs are able to run basic tasks for farm management, including sow cards, working lists, and a general production summary. However, these programs fail when more sophisticated reports are needed, including the specific analytics related to the type of gestation loses, repeat breeders, pre-weaning mortality patterns or calculation of nonproductive days. A major limitation of most of the existing software packages is the inability to create new variables which is an extraordinary limitation when new concepts or problems arise and must be properly analyzed and integrated in to the production system. Most software programs were also designed for single farm use, not allowing the merging of data from different farms. In addition, farmers and veterinarians are not adequately trained on how to use and maximize data management systems.

## The Five Steps in a New Swine Management System

In general, swine data management systems able to meet all the needs of the producers and consultants, have been uncommon in the industry. The idea of having a specific software, mainly for reproductive sows, has been widely accepted in the sector for many years. Some services were offered in this field from the early 1990s, including data entry, benchmarking and descriptive analytics. Having different software packages in the same company was not unusual, generating a problem of coherency since each was performing its proper and on-time distribution of the reports to every role and the information flow to decision making was not optimum.

**Figure 1.**
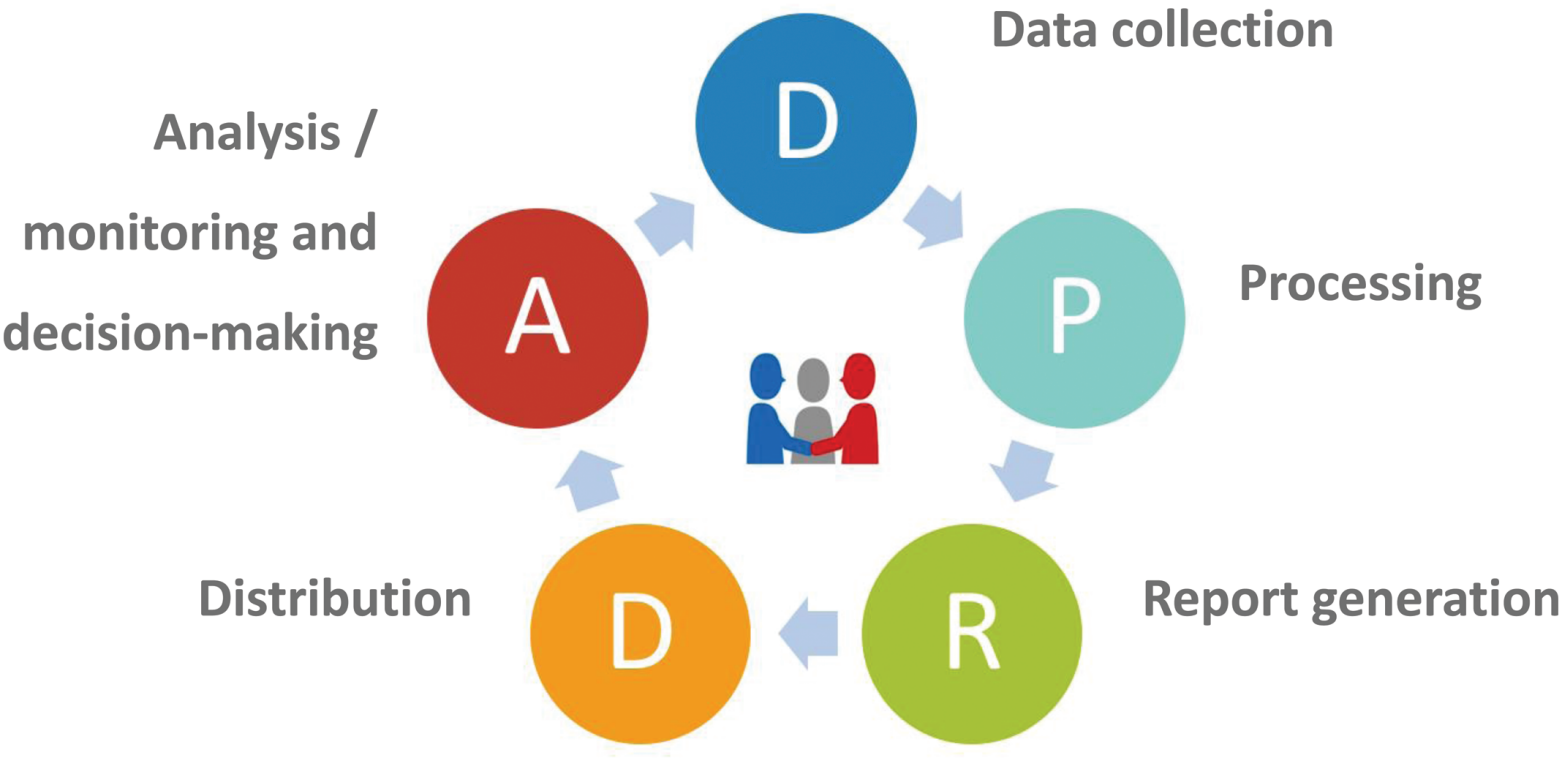
The five steps of an information system.

Based on experience in the last three decades, the authors have defined a swine management system as “*A system made up of tools (software and devices) that together with a working protocol and procedures, including the roles of users, can generate the necessary information to diminish the risk and uncertainties in decision-making*.” This system has five steps (Figure 1), independent of the size and characteristics of the company that uses it.

### Step 1: Data collection

Data are the raw material of the system and can come from human inputs or sensor-robots. Until now, data consisted only of numbers, but the sector is coming closer to the use of images (disease detection based on altered movement patterns, organs, and tissue lesions for presumptive disease diagnostics, smaRt Suite Ro-main, Inc., Quèbec, Canada) and sounds (respiratory distress detected by Sound talks).

### Step 2: Data processing

Data processing is related to the manipulation of data, including several tasks such as validation, sorting or aggregation, management of outliers and missing data. The objective is the correct set-up of databases that allows proper information generation, overcoming interoperability problems (data sharing across systems).

### Step 3: Reporting

Producing the type of reports needed for the farm or company at every level is a major task. From sow cards or working lists (e.g., sows to be mated or vaccinated) up to multivariate regression analysis to define the optimum value for a certain key performance indicator (e.g., age at first mating considering several variables), every farm or company must decide the information needed from every work level (farm staff, farm manager, veterinarian, technical manager, board of directors, or chief executive officers), not forgetting that this could be either technical, economical, or a combination of the two.

### Step 4: Distribution

The objective of this step is sending the right information to the right person at the right time. This step is not properly performed in many cases and is an overlooked reason for data underuse. Sometimes information arrives late and is useless (i.e., hypo-productive sows to be culled if report arrives after mating), or it is too complex for farm staff or too basic for veterinarians or managers. User preferences must also be considered and can include various types (electronic files, text messages, or web applications).

### Step 5: Analytics and decision making

Information must be readable and understood by the recipient, and the recipient must have sufficient time to make key decisions. Until now, analytics were aimed at being mainly explanatory, but due to the amount of quality data available, predictive analytics is becoming a key step. The use of artificial intelligence such as machine learning (an application that provides systems with the ability to automatically learn and improve from experience without being explicitly programmed) or artificial neural networks (an information processing paradigm that is inspired by the way biological nervous systems process information) is expanding.

Following these five steps will establish a robust information system that supports both production efficiency and the required quality standards.

## The Need for New Technologies

In the last decade, the productive global framework has been changing. New information and communication technologies have been developed in all sectors and are reaching livestock production systems. These include wireless connection (3G/4G, Wi-Fi, satellite), powerful mobile devices (smartphones and tablets), sensors and cloud computing. In this scenario, data generation, processing, and use is easier than ever. Moreover, producers are becoming aware that their competitiveness depends on using data appropriately to support decision-making, both for daily decisions as well as strategic decisions.

Modern swine genetics demand a higher degree of understanding of their capacities to optimize performance under commercial conditions. Data capture of the adaptation period of gilts, age for the first mating, optimization of lifetime performance (Iida et al., 2017), causes of early culling, quality of piglets (small or intrauterine growth retarded piglets), mortalities and mortality patterns (Tani et al., 2017), as well as feeding and feeding patterns (Koketsu et al., 1996a, 1996b) are paramount to maximizing the animal’s potential. Without proper use of data generated at the farm, it is difficult to extract its full potential. These data are of great interest to genetic companies which can use it in their selection procedures in a more efficient way.

Quantitative data should not be the only focus since data on quality criteria are a growing component of competitiveness. Quality must be guaranteed within the production chain of live animals including requirements such as piglets with adequate weight, homogeneity suitable for fattening, free of certain diseases or from antibiotic treatments, ensuring welfare status and certain feeding practices (e.g., vegetable only diets). This high demand for both high efficiency and quality cannot be achieved in a production model as complex and sophisticated as the current swine production system without adequate use of the information generated.

## Precision Livestock Farming

As described by Wathes et al. (2008), the concept of “Precision Livestock Farming,” can be defined as “*the management of livestock production using the principles and technology of process engineering*,” and is the principal means by which “smart” sensors or robots will be used in livestock farming. Precision livestock farming is also known as “integrated management systems” and is based on the automatic monitoring of livestock and related physical processes.

This concept addresses some of the shortcomings in data generation and processing and has converged with the global trend toward the digitalization of many products and services. Development of the Precision Livestock Farming concept allowed a very different scenario to generate benefits from the information generated in the sector in the last five years.

## Model-Based Monitoring

**Figure 2.**
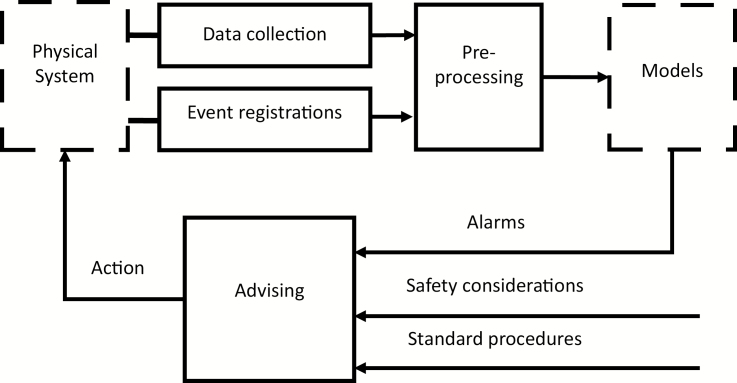
The basic idea of a model-based monitoring system (Jensen, 2016).

The concept of model-based monitoring is illustrated in Figure 2. As Jensen (2016) described, sensors collect data from a physical system with regard to diagnoses, relevant health status or behavior. After collection, this raw data (without processing) feeds several models that should be capable of raising alarms concerning events that have already occurred (detection) or are likely to happen (forewarning). These alarms can then be combined with standard operating procedures to advise the farmer about what action to take.

Data usually collected in precision livestock farming systems can be divided into two categories, namely animal-oriented data and environment-oriented data. As Jensen (2016) summarized, animal-oriented data refers to quantitative measures of the animals’ behavior or their physiological traits, such as animal growth, diseases, behavior, or reproduction. Environment-oriented data refer to quantitative measurements of the environment to which animals are exposed, such as air temperature, relative humidity, water flow or emissions of pollutant gases. To date, attention has focused on the study of individual processes, animal or environmental data, with limited consideration of their interactions. In this regard, connected sensors are becoming less expensive and individual animal and environmental data can be more easily collected through multiple connected devices, which allows accurate real-time analytics and consequently improved decision making.

## Animal-Oriented Data

Animal-oriented data can be collected either by humans, the most important source until now, or by robots, mainly from electronic feeding systems. Other sources are also appearing in the market. These are, for example, images that can be processed and analyzed for different purposes, including disease detection, behavior, and weight calculations.

### Data collection performed by humans

Reproductive data have been the main data source traditionally collected (Tokach et al., 1992; Koketsu et al., 1997a, 1997b). In this regard, the first data collection systems, which were promoted by universities (such as PigCHAMP software: [*P*ig *C*omputerized *H*ealth *a*nd *M*anagement *P*rogram] created originally by the University of Minnesota), generated robust products with a strong technical component. For instance, they allowed improvement in the understanding of some key performance indicators not used to that moment (such as weaning to first service interval, types of abortions, estrus repetition, or types of preweaning mortality). Another characteristic of this initial data collection system was the possibility of generating services around the software package. These included data entry service or bureau service, benchmarking, and the prioritization of farm care. Also, the publication of first-class quality scientific papers based on large databases allowed scientists to describe and explain different effects and set standards for the first time. The results obtained after the first analysis of a big data set conducted by the University of Minnesota showed the extraordinary potential that was hidden in large databases beyond the analysis of individual farms or small groups (PigCHAMP DATASHARE, 1996, 1997, 1999, 2000).

Furthermore, being an academic institution, information was available to the public, which is not usually offered by private companies. Until then, the only companies that had similar systems were the genetic companies, which obviously use their data for company purposes rather than in the interest of the farmers. Occasionally, some finishing data, mainly related to productive performance and health, is used. In this respect, it is important to mention that health data (disease prevalence and treatments) was collected in a generalized manner (total mortality rate in a group of piglets of finishing pigs), but without specifying causes or timepoints which allowed the collectors to know the dynamic of each of the diseases.

### Data collection performed by robots and sensors

#### Estrus behavior. 

Data collected from sensors regarding reproduction has recently been presented to detect the best time for insemination of sows. In this context, several studies have shown that the ideal time for sow insemination is 24 h before ovulation (Soede et al., 1995; Nissen et al., 1997; Almeida et al., 2000). However, the variability between animals is large, and even though estrus usually lasts between 40 and 69 h, it can be as short as 24 h (Soede and Kemp, 1997). Moreover, the detection of estrus relies on visual observations such as a red and swollen vulva, mounting behavior, characteristic growl, nervousness, mucus discharge, and loss of appetite (Bonneville, 2002). Combining sow variability and the difficulty of estrus detection, with the purpose of maximizing fertility, pig producers generally inseminate once every 24 h while the sow shows symptoms of estrus. This method usually delivers good results, provided that good estrus detection has occurred. However, it requires the presence of skilled individuals and multiple doses of semen (often two or even three inseminations per estrus). As Labrecque and Rivest (2018) recently described, PigWatch is a computerized artificial insemination management system designed to predict the best time to inseminate recently weaned sows. This system can determine optimal timing for insemination based on behavior analysis of sows. It consists of motion sensors installed on the top of every stall in the breeding area, a data analysis module and a software user interface. Motion sensors allow continuous and nonintrusive monitoring of sow behavior by assessing its real-time level of activity.

**Figure 3.**
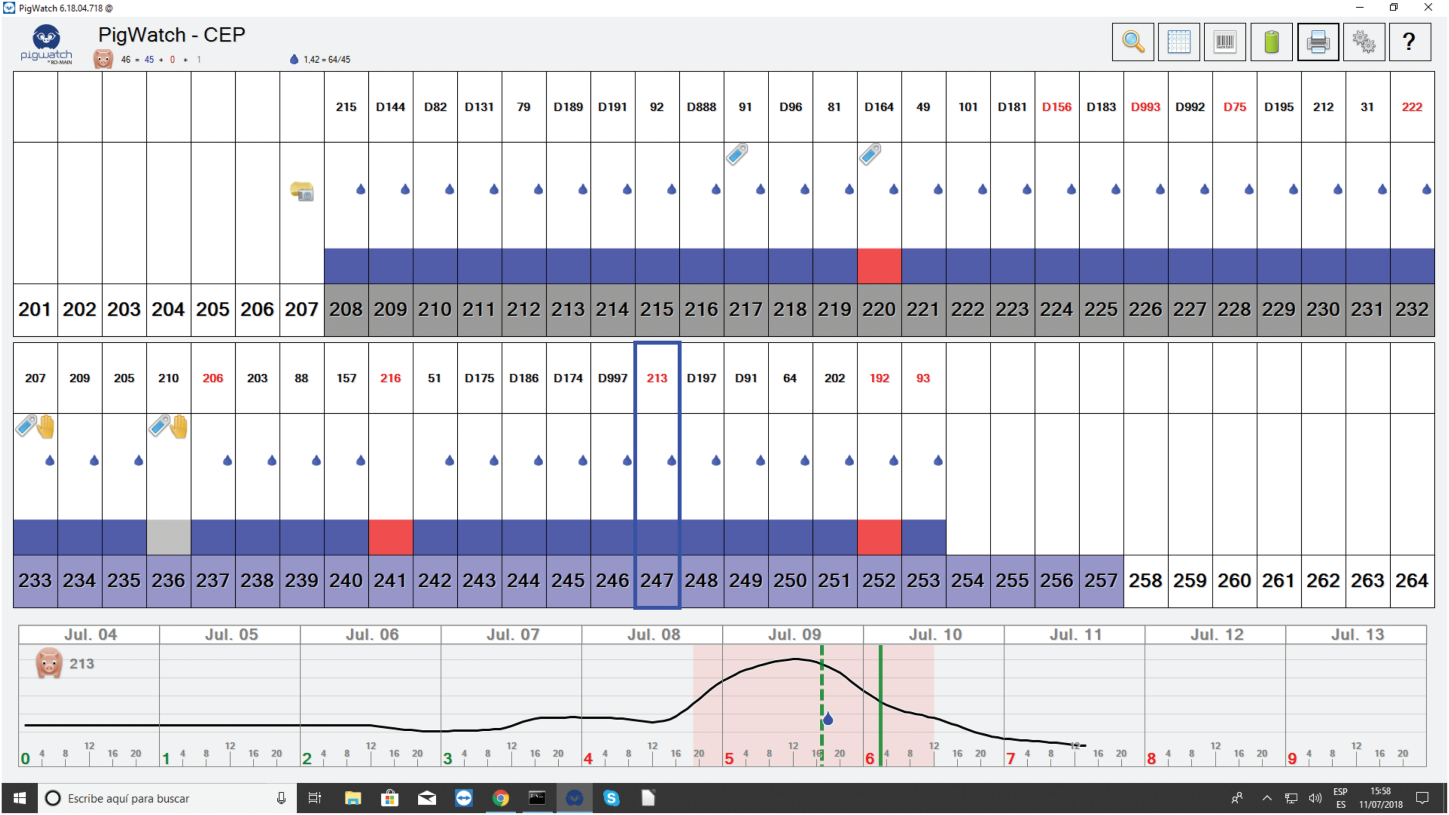
Screenshot of control software for the PigWatch system. It shows sows already inseminated sows with one dose (blue drop), estrus evolution of the selected sow number (213) and the optimum recommendation for breeding (vertical green bar) or the preventive insemination, if not possible to inseminate at the recommended time (vertical green spotted bar). “Hands” in sows 207 and 210 shows manual recommendation of insemination since the pattern is not clear for the algorithm.

Furthermore, as is already known, the behavior of each sow is slightly different. Therefore, the first 2 days after weaning are used to learn about the normal behavior of animals when they are not in estrus. The algorithm looks for a significant increase in activity, which is characteristic of estrus (Figure 3). As behavior data are collected, the algorithm analyzes the pattern of activity to predict the best moment to breed. Once the insemination is completed, the worker registers it in the software by triggering a switch on the sensor and all insemination request indicators disappear.

This system is designed to be installed on commercial farms and has the potential to decrease dependency on skilled labor, improve reproduction, optimize the best use of the boars, and accelerate genetic improvement. Recent studies (Labrecque and Rivest, 2018) used specialized algorithms that consider both sow behavior and worker observations to predict the best timing for insemination while maintaining good reproductive performance. These results proved how big data combined with artificial intelligence algorithms even under commercial conditions, can be transformed into useful information to improve decision making on pig farms.

#### Eating behaviors in gestating sows. 

Other noteworthy variables, which can be measured on pig farms, is the eating behavior of sows. This behavior is usually monitored using an ear transponder with radio frequency identification (RFID) that identifies the individual animal at each visit to the feeder (Bornett et al., 2000). Slader and Gregory (1988) used a combined feeding/weighing device that recorded the time of feed, amount consumed, and the live weight of individual pigs housed in a group. However, in modern systems, pregnant sows are group-housed and fed individually with electronic sow feeder systems. Thanks to these technologies, it is possible to collect enough information to characterize the eating behavior of individual animals (e.g., amount of feed consumed, time spent eating, or preferred time to eat for each sow). For example, most electronic sow feeder systems (Jyga Technologies-GESTAL, Nedap, Schauer, Osborne, Mannenbeck, Asserva, or ACEMA systems-Skiold) present in the market recognize the individual sow using RFID transponders and feed her according to her specific feeding plan by adapted feeding curves.

Besides accurate feeding, the main feature of electronic sow feeder systems is helping farmers to overcome the problems they face in group-housed pregnant sows such as the competition between animals, stress (especially in gilts and submissive sows) and waste of feed. Moreover, electronic sow feeder systems allow sows to reach the ideal body weight condition for farrowing, a reduction of time spent on feeding, and above all the rapid detection of sows with a deviated feed intake pattern which could be an indicator of disease. All these systems generate alerts when a sow is not eating but usually do not go beyond this. A recent publication (Iida et al., 2017) demonstrates that more subtle variations in feed intake of gestating sows can be detected and related to their gestation losses since sows losing gestation time visit the feeder fewer times and tend to eat less. The same authors demonstrated differences in the patterns among parities and genetic lines. By the proper implementation of these algorithms, abnormal eating behaviors can be detected in advance before the sow stops eating completely, where the situation will be likely more severe.

#### Eating behaviors in lactating sows. 

**Figure 4.**
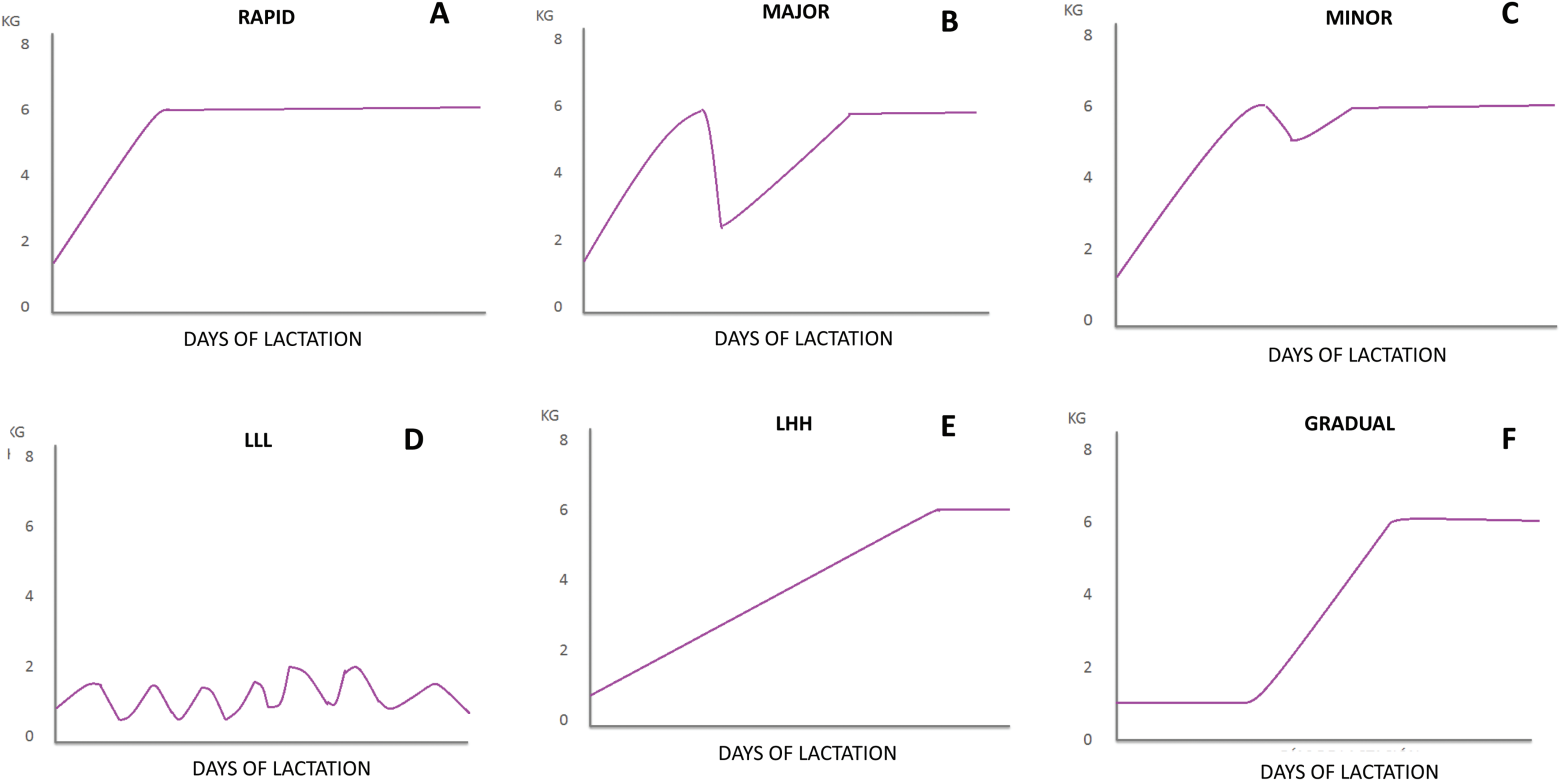
Feed intake patterns of lactating sows described by Koketsu et al. (1996b). (A) rapid increase in feed intake; (B) major and (C) minor drop; (D) low feed intake throughout lactation (LLL); (E) low intake during the first week then an increase in feed intake for the remainder of lactation (LHH); and (F) gradual increase.

**Figure 5.**
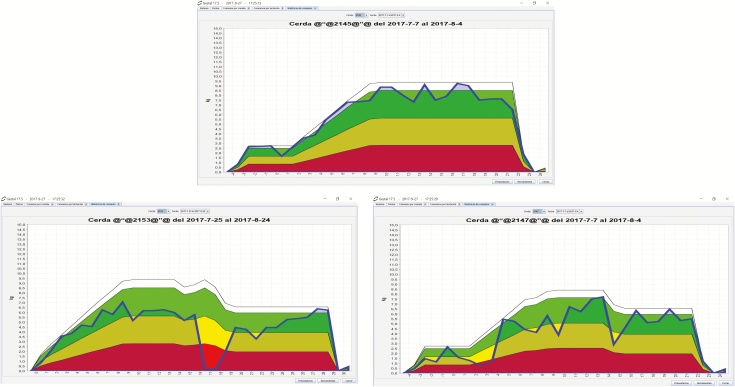
Graphs of feed intake of lactating sows obtained by the electronic sow feeder system (GESTAL SOLO, JYGA Technologies). (A) Normal feed intake pattern, (B) major drop and (C) low feed intake throughout lactation. Green space shows the ideal overtime intake for that particular sow. Yellow space shows a warning since intake its reduced. Red space shows a strong deviation and therefore high risk of later reproductive impairment.

Similar electronic sow feeder systems are also available for lactating sows, which are individually housed. All these systems allow the producer to decide and adjust the amount of feed delivered to each sow. New options allowing the sow to choose how much and when to eat have recently arrived at the market (Gestal Solo, JYGA Technologies), thus enabling the farmer to know the lactation intake pattern. These data are very relevant since deviation from the ideal feed intake pattern can impair the productive performance of sows. Koketsu et al. (1996b) categorized the lactation feed records of more than 25,000 lactating sows on 30 commercial farms in six patterns (Figure 4): 1) rapid increase in feed intake; 2) major and 3) minor drop; 4) low feed intake throughout lactation; 5) low intake during the first week then an increase in feed intake for the remainder of lactation; and 6) gradual increase. In this study, multiple regression analyses revealed that average daily feed intake of sows during lactation had linear or nonlinear associations with the key performance indicators of swine production. We demonstrated that sows having either a lower feed intake throughout complete lactation or having a major drop during the first week, had longer weaning-to-first service interval and weaning-to-conception and had lighter litter weight at weaning than those with rapid increase, minor drop and gradual increase in feed intake. Until now these results were very difficult or almost impossible to track in commercial farms. These feed intake patterns described by Koketsu et al. (1996b) have recently been confirmed by Piñeiro et al. (unpublished data) thanks to the use of an electronic sow feeder system (Gestal Solo, JYGA Technologies). Figure 5 shows the graphs of three patterns of feed intake recently obtained from commercial farms previously described by Koketsu et al. (1996b): the “normal pattern” (rapid increase of feed intake during lactation) and the two patterns which deviate most from the ideal intake pattern and which can impair the productive performance of sows (major drop and low feed intake throughout lactation).

Finally, electronic sow feeder systems are also available for growing-finishing pigs (Nedap, Acema, and Schauer). As in group-housed sows, these systems will recognize individual growing pigs via an RFID transponder, but in this case, the system will allow the pigs to be fed ad libitum. Combining data of feed and animal body weight, the breeders can select those pigs which utilize feed most efficiently. The principal advantage of electronic sow feeder systems is the possibility of massive data collection, detecting deviations from standards, early alerts on disease or in combination with other data (i.e., health) to generate useful insights with minimum effort.

#### Early disease detection based on image analytics. 

A motion-based video system for early disease detection has recently been described (Fernández-Carrión et al., 2017). These authors detected a significant decrease in the motion of pigs 4 days after experimental infection with the African swine fever (ASF) virus in wild boars, the same day that the virus was detected in blood using qPCR, and 3 days before clinical signs of ASF were observed. These results illustrate the potential of video image processing for early detection of ASF and other infectious diseases although they must be confirmed at a commercial scale since other factors (i.e., stocking density or temperature) could affect the natural expression of motion in pigs.

## Environment-Oriented Data

### Environmental farm control equipment

Environmental stress produced by variations in temperature, humidity, or gasses has been largely described in pigs making them susceptible to triggering or worsening health problems. Thus, continuously low temperatures (18–20°C) increase the frequency of diarrhea (Le Dividich, 1980; Feenstra, 1985). Cold temperatures, increase susceptibility to colibacillosis (Armstrong and Cline, 1977) and increase the risk of Actinobacillosis by combining low temperatures and low humidity (Kreukniet et al., 1990). Finally, high humidity increases the risk of streptococcal disease (Dee et al., 1993). To minimize these effects, environmental control systems are used, but they are not perfect. These systems require maintenance, adjustments and supervision that are not always perfectly done, and therefore, higher variations than expected in the control of these key performance indicators occur on farms, increasing the risk of health problems.

Little use of these data are routinely made beyond alerts (values above or below a certain threshold) and usually no records are kept, and therefore no value can be extracted. When a problem regarding environmental control is suspected, only portable data loggers, mainly for temperature, are placed in specific barns, and then a manual comparison with health indicators (normally mortality or treatments administered) is performed. This approach is only explanatory and of limited value, since it affects only specific situations and not a routine.

There is limited literature citing combined animal and environmental data with the purpose of strategic decision making. As mentioned, this is probably because the amount of environmental data at large scale is a relatively new phenomenon. Recent research by Jensen and Kristensen (2016) has addressed this issue. These authors recorded temperature data at the pen level and were able to use this information to predict pen fouling and diarrhea up to 3 days before these events occurred.

**Figure 6.**
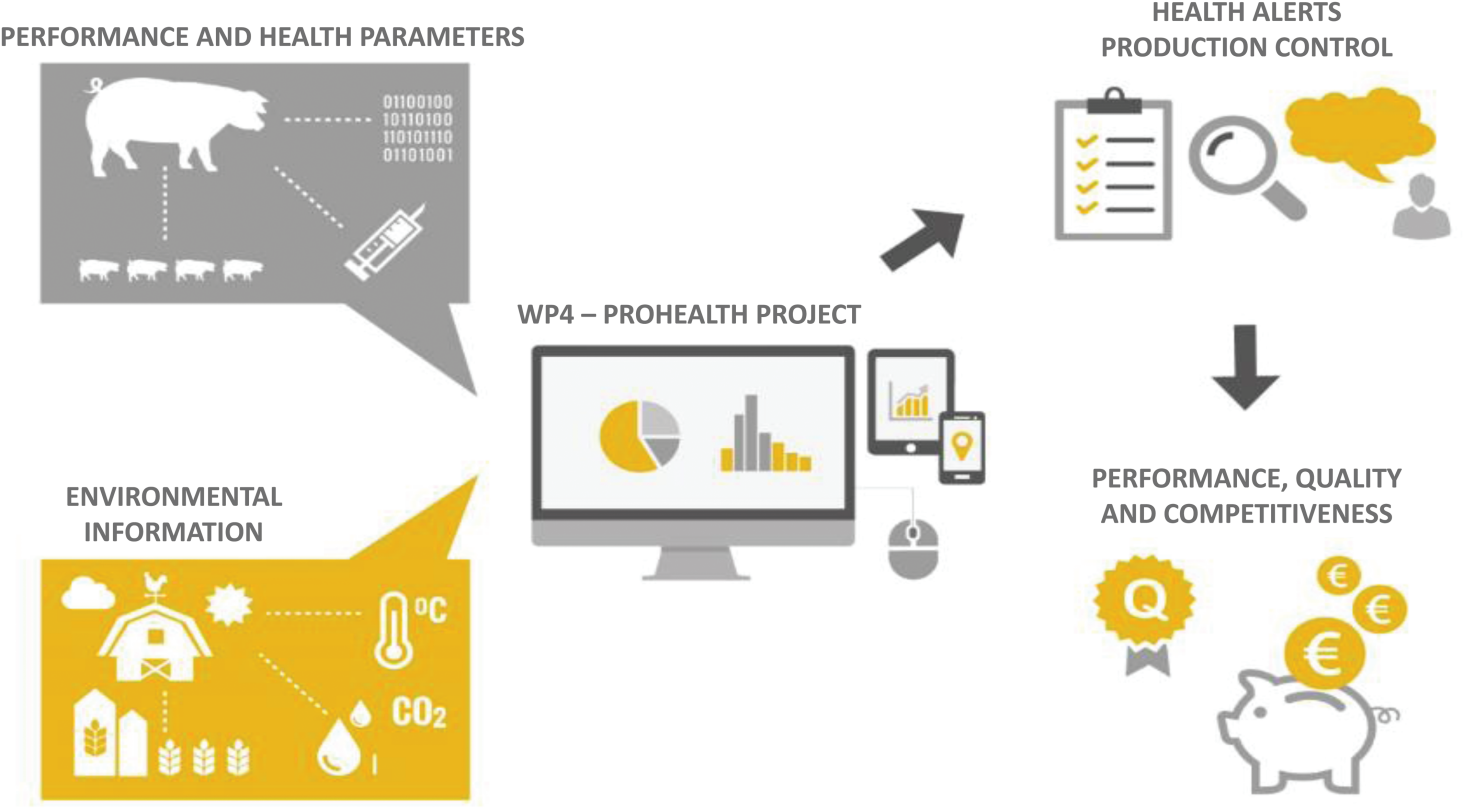
Scheme for working system to monitor developed in PROHEALTH project. Research based on EU-FP7/funded PROHEALTH-project (no.613574).

One of the main factors which influence outbreaks of respiratory disease is the environment the pigs inhabit. Thanks to the development of new technologies, farmers are now able to continuously monitor, air quality, temperature, and humidity in real time via sensors. The high variability of these environmental parameters could be risk factors favoring the development of respiratory diseases (Stärk et al., 2000). Also, in this way, the PROHEALTH project (Research based on EU-FP7/funded PROHEALTH-project [no.613574]), showed how big data could be used to fight diseases (Figure 6). Certain animal diseases can be triggered by changes in barn conditions. Small and inexpensive sensors were used to monitor environmental variables in the pig house and health data (treatments, mortality, and euthanized pigs) were collected at the same time from 59 batches and almost 15,000 nursery and finishers from EU farms for 15 months. A GRU-autoencoder (a type of neural network built using deep learning), can learn to reconstruct raw sensor data about factors that may or may not lead to an increase in respiratory disease prevalence in pigs. This system out-performed state-of-the-art disease alert techniques and showed that a change in the environment in which the pig lives, measured by sensors, can indicate an increased number of pigs showing symptoms 1–7 d in the future.

### Real-time biosecurity control

Biosecurity is defined as the implementation of measures that reduce the risk of disease agents being introduced and spread on farms (FAO, 2010). Improving the level of biosecurity is considered to result in limited introduction and spread of disease, resulting in reduced morbidity and mortality rates, making biosecurity a tool in disease eradication programs as well as in daily health management.

To date, most biosecurity program measures are based on scoring systems or survey forms. For instance, researchers from Ghent University developed a scoring system called Biocheck UGent(Laanen et al., 2013; Postma et al., 2016) as a risk-based scoring tool to evaluate the biosecurity quality of pig herds. The scoring system was generated through expert opinion panels. Another scoring system has been developed by the University of California-Davis (Disease Bioportal) for dynamic risk assessment. Farm benchmarking is also based on surveys. Nevertheless, it is important to keep in mind that perception is a subjective aspect. Therefore, opinion-based scoring systems used as a tool to control biosecurity are not the best option. Allepuz et al. (2018) recently indicated the need to develop more complex models to provide a quantitative risk assessment, emphasizing that this kind of model could be more precise in mimicking reality and might give a more accurate estimation of the probability of virus spread within herds. The same authors noted that these quantitative models could not be developed easily because of the lack of relevant long-term data. Moreover, according to Sternberg-Lewerin et al. (2015), it is difficult to obtain quantitative data from field studies. In contrast, the role of people in disease transmission has been carefully studied over the last decade. People can carry viruses in their nasal mucosa without being infected. They can also be infected and shed pathogens as healthy or as ill carriers. Movement of people between barns, pigs, and fomites, has been recently defined (research based on EU-FP7/funded PROHEALTH-project (no.613574)) as the most important factor affecting internal biosecurity.

A new approach addresses this issue by using real-time devices (B-eSecure System) to control the internal movement of farm staff. Movements are qualified depending on the health status of every barn, defined by PCR to specific diseases, being “safe” from PCR(−) to PCR(+), “unsafe” between PCR(+) and risky from PCR(+) to PCR(−). The system is based on a small *Bluetooth* transmitter (called *Beacon*) which each worker carries during farm work. Readers are installed at every barn access, including lockers and showers. Data are sent to the internet real-time and based on cloud processing, and the system generates real-time control and alerts based on the pattern of movement of the workers. Based on its use, it has been possible to decrease viremia on farms (Díaz et al., 2018), by reducing risk movements, improve health and performance (Geurts et al., 2018) and even predict the number of piglets weaned depending on the percentage of reduction of risky movements (Arruda and Allen, 2018). This new approach is very relevant since it generates data where previously there was none and can be used either as simple daily health controls to generate more sophisticated explanatory or predictive models that can help to control the main risk factors affecting internal biosecurity.

## Conclusions and Implications

The digitalization process that includes software, devices, systems, standard operating procedures, analytics, and communications is ongoing in the swine sector and enables the collection and use of large quantities of data. This phenomenon has already brought great advances in the concept of precision livestock farming. Further steps in this digitalization process will improve production efficiency, health, and welfare on farms under the quality standards that modern production requires. In the next few years, this new digitalization process will generate new knowledge in most of the relevant topics in swine production including nutrition, health management, reproduction, genetics, biosecurity, behavior, welfare, and even pollutant emissions. This will bring unprecedented changes and advantages to the industry and huge opportunities for professionals in the global swine production sector.

## About the Authors

About the AuthorsCarlos Piñeiro earned a DVM at the Complutense University of Madrid, a PhD from Meiji-Tokyo University, and a Diplomate from the European College of Porcine Health Management. He is a member of the Spanish Focal Group for Digitization and Big Data of the Agricultural and Livestock sector in Spain and he has served as managing director of PigCHAMP Pro Europa SL since 2000. His current research focuses on real time control of biosecurity on farms and control of antibiotic use in pig and poultry production systems.

María Rodríguez earned a MEng in agricultural engineering at the Polytechnic University of Madrid and a PhD at the Polytechnic University of Madrid. She was a project research assistant in the Department of Animal Physiology at Complutense University of Madrid from March 2013 to February 2018. Since March 2018, she has been working for the project management and R&D Department at PigCHAMP Pro Europa SL. Her current work focuses on sustainable usage of trace minerals in animal production programs.

Joaquín Morales earned a DVM and PhD from the Autonomous University of Barcelona and a Diplomate from the European College of Porcine Health Management. Since 2002, he has served as Head of the Applied Research Department of PigCHAMP Pro Europa SL, working as the main investigator in all swine research studies conducted at the company. Since 2018, he has collaborated with the European University of Madrid as an assistant professor in the Master of Veterinary Clinical Studies program.

Yuzo Koketsu earned a BVSc from Obihiro University and MVSc from Osaka Prefecture University. After 12 years of feed industry experiences, he obtained a PhD from the University of Minnesota. This was followed by 2 years of postdoctoral research and then 4 years as a faculty member in the College of Veterinary Medicine at the University of Minnesota. He obtained the Horizon Award of PigCHAMP for his farm data analysis and benchmarking work for the U.S. swine industry. Since 2000, he is Professor in the School of Agriculture at Meiji University, Japan. His current area of research includes analysis of farm data and digitalization of data from swine production systems.

María Aparicio received a DVM from Complutense University of Madrid and has worked at PigCHAMP Pro Europa SL since 2000. She is currently responsible for the Data Management and Analysis Department. Through this department, PigCHAMP Pro Europa markets products and services aimed at supporting decision-making processes from small family farmers to large producers and multinational companies.

Edgar Garcia Manzanilla (DVM, PhD, MPVM, Dipl. ECPHM) obtained his PhD in Barcelona. He moved to the University of California—Davis (USA) where he obtained a Master’s degree in Preventive Veterinary Medicine in data management and analysis. In 2014, he joined the Pig Department of Teagasc, Ireland. His research interests are on monogastric nutrition and reduction of antimicrobial use in farms from an epidemiologic point of view.
